# Our Experience With the Correction of Prominent Ear Deformity

**DOI:** 10.7759/cureus.19772

**Published:** 2021-11-20

**Authors:** Farkhandah M Iqbal, Touqeer Hussain, Yusra Afzal, Mirza Shehab A Beg

**Affiliations:** 1 Plastic Surgery, Liaquat National Hospital and Medical College, Karachi, PAK; 2 Plastic and Reconstructive Surgery, Medicare Clinics, Karachi, PAK; 3 Plastic and Reconstructive Surgery, Liaquat National Hospital and Medical College, Karachi, PAK

**Keywords:** ear reconstruction, cosmetic ear surgery, chongchet technique, otoplasty, prominent ears

## Abstract

The ear has a unique architecture of cartilage and skin. The incidence of the prominent ear is about 5%. Surgical correction of the prominent or protruding ear can be carried out either by anterior or posterior approach. We created antihelical fold of cartilage by utilizing a posterior incision to score the anterior cartilage of the lateral scapha with a knife. Sutures were often used to uphold the produced fold. The additional procedure of conchal reduction and concho-mastoid suture was done when required. The objective of our research is to evaluate the patient’s and surgeon’s satisfaction with our technique of prominent ear correction and identify any complication if it occurs post-operatively.

This is a retrospective study over a period of eight years (2011-2018) which includes all patients presented to Liaquat National Hospital with prominent ear. A total of 47 patients were included. Patients with a previous history of otoplasty were excluded. Patients were followed up for at least six months postoperatively. The outcome was assessed via Visual Analogue Score by a patient, surgeon, and a third observer (assessor). The average score by the surgeon was 7.9, by the patient it was 8.4 and by the assessor it was 8.1. The average pre-operative concho-mastoid distance was 2.2 cm which decreases to 1.4 cm post-operatively. Correction of the prominent ear by this technique is safe and easy. We did not experience any major complication, giving reproducible and good aesthetic results.

## Introduction

The anatomy of the external ear is very complex. It differs from person to person and between two sides of the same individual. Prominent ear deformity has a great influence on the development of a person’s brilliance and social acceptance. The abnormal position is not the only problem, but its abnormal relation to the skull in its normal position gives a poor image. In the Caucasian population, the incidence of the prominent ear is about 5% and it accounts for the most prevalent congenital head and neck deformity [1]. It is usually isolated without other anomalies or syndromic association, but it may be considered as an aesthetic handicap [2]. With this abnormality, only 8% of patients have a family history [3]. In around 61% of affected children, ear abnormality is found at birth and there is no gender predilection. The anatomical features of the prominent ear are described as: (1) Lack of sufficient anti-helical fold; (2) Outsized deep conchal bowl; (3) Abnormalities of lobules; (4) Inadequate definition of helical rim. All these features are usually present in combination in these patients [4,5].

For the past few decades, there have been various techniques addressing the problem of prominent ears with sutures or cartilage scoring. In 1854, Dieffenbach described his otoplasty technique [6], meanwhile in 1881, Elly wrote a case report on otoplasty technique [7]. Converse, in 1955, presented his technique in which he gave incomplete cartilage incisions from the posterior approach in combination with fixative sutures [8]. In November 1960, Chongchet proposed his technique by using posterior approach to score anterior cartilage of the lateral scapha to create anti-helix [9].

In our cases, we modified Chongchet technique by doing a few additional steps and evaluated patient’s as well as surgeon’s satisfaction post-operatively, and identified any complication if it occurred post-operatively.

## Materials and methods

Patient population

All the patients presented in the outpatient department of Liaquat National Hospital and Medical College from January 2011 to December 2018 with prominent ears, aspiring for aesthetic improvement due to emotional and social setback among peers were included in the study. After routine hematological workup patients were operated on by a single surgeon. Patients with a previous history of otoplasty were excluded from the study. Patients were followed up for at least six months postoperatively. Any complication which occurred during this period was recorded and addressed accordingly.

Analysis of results

Pre-operative concho-mastoid angle is measured by the same surgeon who is going to perform surgery and recorded on paper. Post-operatively, the patients were followed up for at least six months. The outcome is assessed using Visual Analogue Score. The patient, surgeon, and a third observer (assessor) were asked to score the following parameters on a scale of 1-10 with score 1 referring to extremely dissatisfied and on the other end score of 10 indicating extremely satisfied. Parameters, which were accessed, include: Improvement in the natural contour of ears, improvement in frontal view, satisfaction with new antihelical fold, improvement in irregularity at conversational distance, improvement in asymmetry of concho-mastoid angle.

Post-operative concho-mastoid angle was measured and it was compared with pre-operative measurement. Data was analyzed on SPSS version 21.0 (IBM Corp., Armonk, NY).

Operative technique

**Figure 1:**
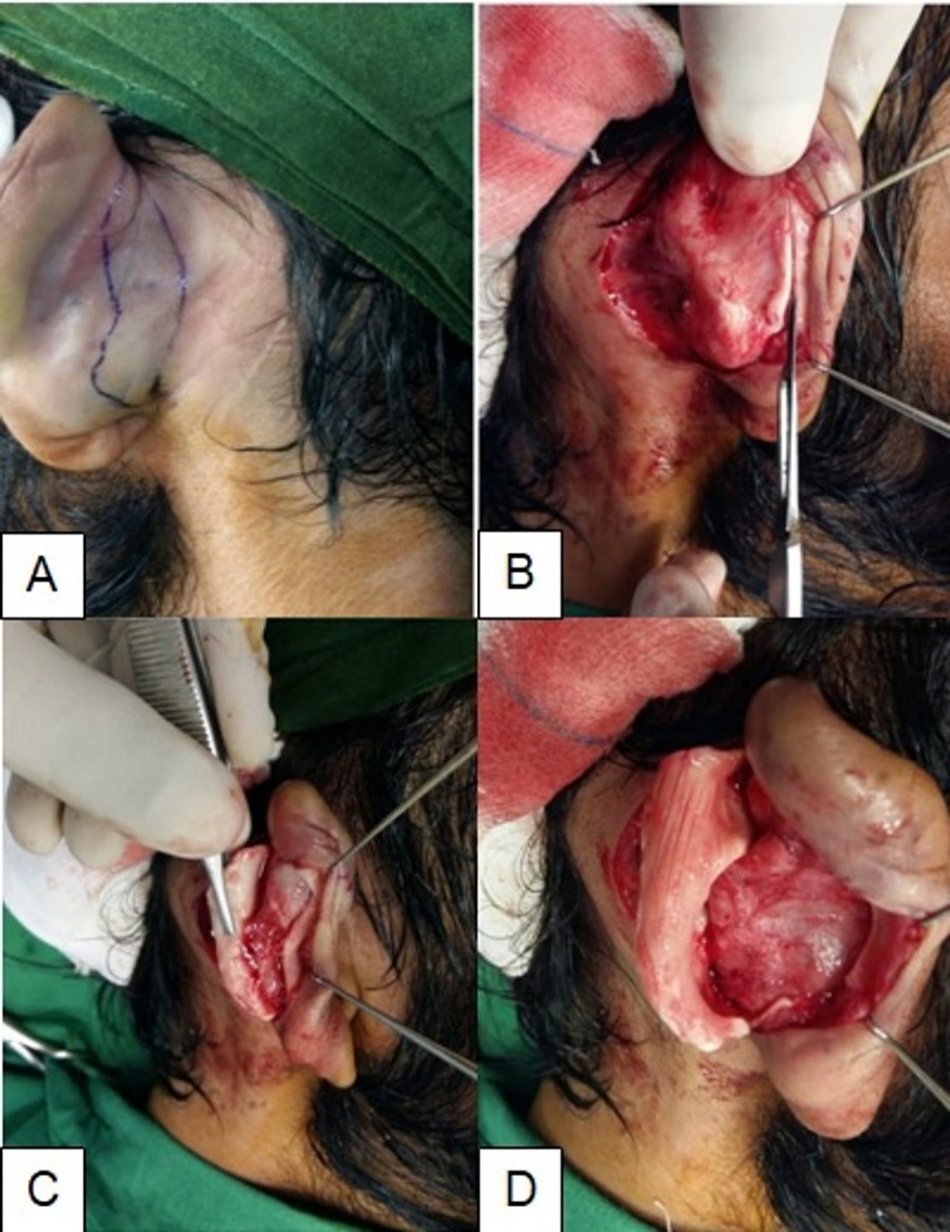
Steps of operative technique. A: Marking of lazy S-shaped cutaneous incision at post-auricular area. B: Incision over cartilage. C: Posterior scoring of conchal cartilage. D: Concho-mastoid suturing.

The surgery was performed in the supine position on both ears at the same time, with the patient under local anesthesia with or without intravenous sedation. Xylocaine 1% with 1:200,000 adrenaline is infiltrated on both the anterior and posterior surface of the ear to yield a painless, bloodless field. This infiltration also aids in undermining due to hydrostatic dissection between the skin and underlying cartilage. The future anti-helix fold is marked with methylene blue dye and deep tattooing done using a 27G needle. A lazy S-shaped cutaneous incision was made at the post-auricular area about 1.5 cm lateral to the auriculo-mastoid sulcus. The careful undermining of skin was done to expose the anterior auricle. The future antihelix (marked already) is scored by numerous longitudinal partial-thickness cuts to create an even antithetical fold. To reduce the conchal bowl, we excised an oval-shaped portion of cartilage from the posterior side of the auricle if necessary. All these steps of the operative technique are shown in Figure 1. A non-absorbable suture (polypropylene 4-0) was applied to fix the concha to the periosteum of the mastoid. The skin was closed by continuous subcutaneous stitches (Polypropylene 5-0). A circumferential crepe-bandage is applied for four weeks which works as a splint. Dressings were changed on regular basis. Skin sutures were removed on the 8th post-operative day.

## Results

**Figure 2:**
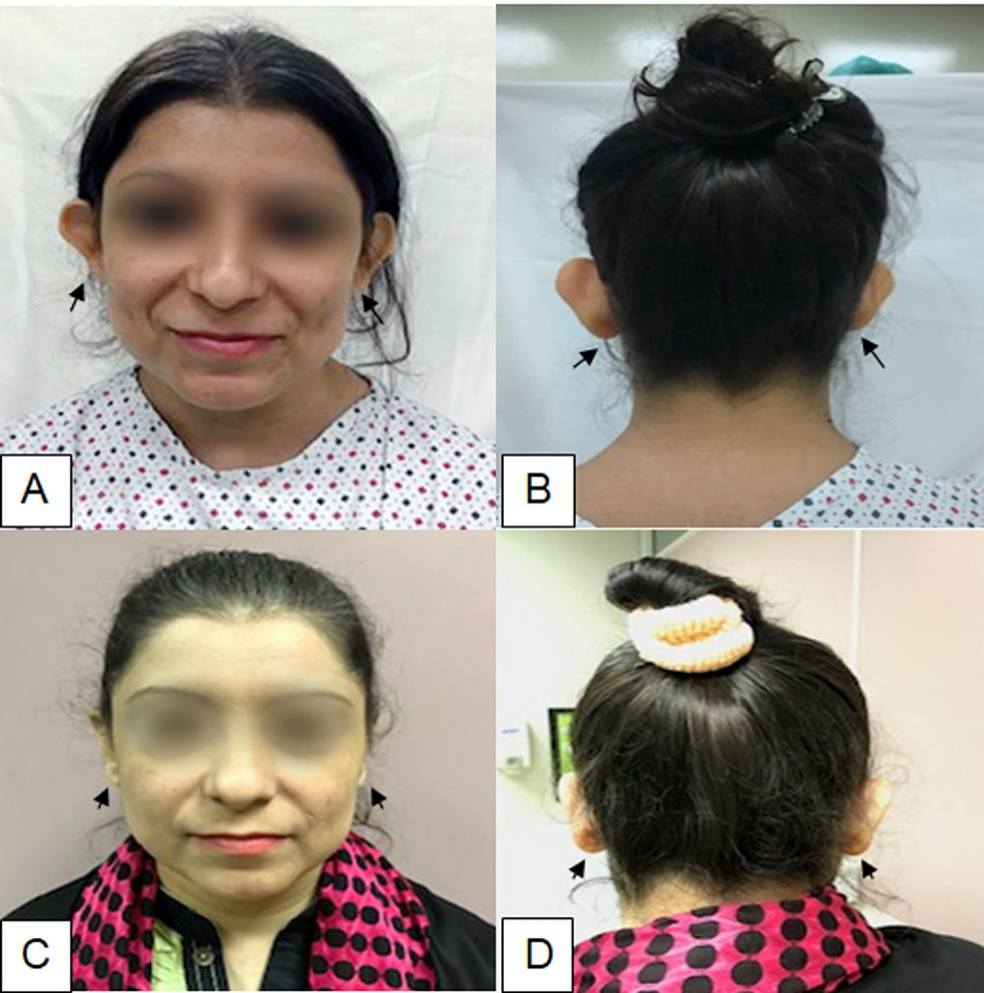
A: Pre-operative frontal view of the face showing prominent ears. B: Pre-operative posterior view of the head showing prominent ears. C: Six months post-operative frontal view of the face showing corrected prominent ear deformity. D: Six months post-operative posterior view of the head showing corrected prominent ear deformity.

The total number of patients who were operated on from 2011 to 2018 were 47, 28 of them were females and 19 were males; mean age of patients is 22 ± 5 years. The average score given on each assessed parameter by the surgeon, patient, and assessor is summarized in Table 1. The average pre-operative concho-mastoid distance was 2.2 cm (1.9-2.5 cm) which decreases to 1.4 cm (1.3-1.6 cm) post-operatively. One (2.1%) patient developed postoperative hematoma which was drained by a stab incision. One (2.1%) patient had palpable cartilage and three (6.3%) patients had palpable sutures but none of them opted for revision surgery. Figure 2 shows the result in a female patient after six months of surgery.

**Table 1:** Visual Analogue Score given by the surgeon, patient and third observer

PARAMETERS		SURGEON	PATIENT	ASSESSOR
1.	Improvement in the natural contour of ears	7.6	8.7	7.9
2.	Improvement in frontal view	8.2	8.4	7.9
3.	Satisfaction with new antihelical fold	8.3	8.8	8.6
4.	Improvement in irregularity at conversational distance	8.4	8.5	8.5
5.	Improvement is asymmetry of concho-mastoid angle	7.4	7.9	8.0
Average total score		7.9	8.4	8.1

## Discussion

Prominent ears are usually not seen together with other congenital problems or mental problems. However, these can result in psychological problems. All these problems arise from the fact that the ears become noticeable although they should not. So, the aim of the treatment is to achieve the most natural outcome and to ensure that the ears are not noticed at first sight from the front. The prominent ear is characterized by increased concho-mastoid angle, deep conchal cartilage, unfolded antihelical fold, or a combination of these [1].

Over the past few decades, there have been various techniques, more than 200, to address this problem [10]. The prominent ear can be treated surgically. It can either be done by suturing and cartilage scoring or both. Surgical correction of the prominent or protruding ear can be carried out either by anterior or posterior approach [8]. In 1854, Dieffenbach was the first to describe his method of otoplasty to correct the post-traumatic protruding ear in a patient [6]. In 1881, Elly described his first case report of otoplasty, for treating the prominent congenital ear [7]. After that many other surgeons described their technique with different modifications, specifically the incision-scoring technique of anti-helix [11].

Furnas described a suture-only technique in which he used permanent sutures to fix concha with mastoid by approaching the cartilage through the posterior incision to expose the auricle [12]. The problems encountered in this technique were secondary recurrence of prominent ears because of warping and irregularities produced in the molding of the conchal floor [10].

Chongchet and Crikelair utilized a posterior incision to score the anterior cartilage of the lateral scapha with a knife to create the anti-helix [9].

In 1960, Mustardè explained the procedure by remodeling the articular cartilage using mattress sutures, there was a 7% relapsed rate because the cartilage was too weak [13]. In contrast, Stenström used a rasp to shape the cartilage through small posterior access [14].

El Hariry et al. did a comparative study between two techniques described earlier by Mustarde and Stenström [15]. In that study, 10 patients were divided into groups A and B. In group A surgical correction was done using Mustarde technique and in Group B using Stenstrom. Their results were satisfactory with no such major complication in each technique, only the irregularity in the helix was found in 5% patients in group B.

We modified the Chongchet technique [9], by adding two more steps to it. We created a natural fold of cartilage using the Chongchet technique by cartilage incision at the border between scapha and antihelix, and anterior scoring of cartilage through a posterior approach. Sutures were often used to uphold the posterior fold. We modified it further by an additional procedure of conchal reduction and concho-mastoid suture. These two steps further improved aesthetic appearance and reduced the risk of complications. Chongchet did his study on a series of 21 patients [9]. A total of 4.76% patients developed infected hematoma [9], while in our technique 2.12% patients developed hematoma. Further in Chongchet’s study, 9.52% cases were under-corrected [9], none of our patients has under-corrected result. Patients were satisfied post-operatively even on six months follow-up visit.

This study demonstrates improvement in outcome and reduction in complication rate with the use of our technique.

## Conclusions

Our technique for correction of aesthetically unpleasant prominent ears is safe as reflected by low complication rate and it is easy to learn. We found good outcome in most of our patients. The majority of our patients were satisfied with the procedure as reflected by their assigned score to aesthetic improvement. We highly recommend employing this technique in other setups as well, where surgical expertise is available.
